# Deficiency in CCR2 increases susceptibility of mice to infection with an intracellular pathogen, *Francisella tularensis* LVS, but does not impair development of protective immunity

**DOI:** 10.1371/journal.pone.0249142

**Published:** 2021-03-24

**Authors:** Sherry L. Kurtz, Roberto De Pascalis, Anda I. Meierovics, Karen L. Elkins

**Affiliations:** Laboratory of Mucosal Pathogens and Cellular Immunology, Division of Bacterial, Parasitic and Allergenic Products, Center for Biologics Evaluation and Research, U.S. Food and Drug Administration, Silver Spring, MD, United States of America; Rutgers Biomedical and Health Sciences, UNITED STATES

## Abstract

CCR2 is the major chemokine receptor that regulates appropriate trafficking of inflammatory monocytes, but the role of this chemokine receptor and its ligands during primary and secondary infection with intracellular infections remains incompletely understood. Here we used murine infection with the Live Vaccine Strain (LVS) of *Francisella tularensis* to evaluate the role of CCR2 during primary and secondary parenteral responses to this prototype intracellular bacterium. We find that mice deficient in CCR2 are highly compromised in their ability to survive intradermal infection with LVS, indicating the importance of this receptor during primary parenteral responses. Interestingly, this defect could not be readily attributed to the activities of the known murine CCR2 ligands MCP-1/CCL2, MCP-3/CCL7, or MCP-5/CCL12. Nonetheless, CCR2 knockout mice vaccinated by infection with low doses of LVS generated optimal T cell responses that controlled the intramacrophage replication of *Francisella*, and LVS-immune CCR2 knockout mice survived maximal lethal *Francisella* challenge. Thus, fully protective adaptive immune memory responses to this intracellular bacterium can be readily generated in the absence of CCR2.

## Introduction

Many intracellular pathogens, including the facultative intracellular bacterium *Francisella tularensis*, preferentially invade and replicate in host macrophages [1]. Host responses to intracellular infections involve interactions between macrophages with many other responding cell types, such as lymphocytes responsible for adaptive immune responses and memory. The movement of inflammatory monocytes between and within tissues is orchestrated in large part by the chemokine receptor CCR2 and its ligands.

CCR2 is expressed as two splice variants on the surface of a variety of myeloid cells, including monocytes, macrophages, some dendritic cells, and basophils, as well as lymphoid cells such as natural killer cells and some CD4^+^ Th1 T cells [2]. A key function of CCR2 is enabling the rapid movement of inflammatory monocytes out of the bone marrow into the peripheral blood and tissues in response to inflammation and infection [3]. Moreover, although many chemokine-receptor interactions are redundant, monocyte recruitment appears to be unique to CCR2 [4]. CCR2 is engaged by several chemokines produced in response to inflammation. In mice, the known CCR2 ligands include the monocyte chemoattractant proteins (MCPs) MCP-1/CCL2, MCP-3/CCL7, and MCP-5/CCL12 [2]. Of these, MCP-3/CCL7 can also signal through a different chemokine receptor, CCR3. MCP-3/CCL7 and especially MCP-1/CCL2, which binds CCR2 uniquely, have generally been viewed as the major murine ligands for CCR2, particularly *in vivo* [2,3,5]. In both humans and mice, these chemokines are produced not only by myeloid cells but also by a variety of others, including endothelial, fibroblasts, epithelial, smooth muscle, and microglial cells [2,5].

Human genetic polymorphisms in gene alleles for CCR2 and its ligands have been associated with differential susceptibility to a number of infections of people, including malaria, HIV, Chagas disease, and *Mycobacterium tuberculosis* [6]. In animal models, deficiencies in CCR2 and some of its ligands alter susceptibility to several experimental infections, including those caused by intracellular pathogens [7]. Among the best studied of these is *Listeria monocytogenes*. CCR2 knockout mice (CCR2 KO) are quite susceptible to intravenous (IV) *L*. *monocytogenes* infection: compared to wild type mice, CCR2 KO mice exhibit greatly increased bacterial burdens in spleens and livers, increased pathology in livers that includes multifocal inflammation and necrosis, and death at low bacterial doses [8–10]. On the other hand, complete CCR2 deficiency has different impacts on murine *M*. *tuberculosis* infection that vary with the route of infection and bacterial strain used [11–13]. Thus, the relative importance of CCR2 in primary intracellular infections remains an open question, and its role in secondary challenge with an intracellular pathogen, following survival of a first infection or following vaccination, has not been evaluated.

We [14,15] and others [16] have used murine infection with the Live Vaccine Strain (LVS) of the facultative intracellular bacterium *F*. *tularensis* as a model to uncover mechanisms of protective immunity to intracellular bacteria. The major features of LVS infection of inbred mice, including intramacrophage replication and the importance of Th1 T cells, have much in common with many intracellular pathogens [14,15,17]. Further, the outcome of LVS is route-dependent: LVS administered to mice intradermally (ID) establishes a sublethal infection, while LVS administered to mice intraperitoneally (IP) or IV is lethal, and intranasal (IN) infection is intermediate. Moreover, ID LVS infection provides very strong protection against subsequent lethal IP LVS challenge. Thus LVS serves as an intracellular infection model in mice that allows both vaccination and challenge with the same bacterium, while simultaneously modeling human vaccination against *Francisella* and other intracellular bacteria.

In previous studies of *Francisella* infections, splenic monocyte populations did not expand quickly in CCR2 KO mice given LVS IV, and spleens had higher bacterial burdens within the first day of infection [18]. Consistent with the role of CCR2 found in host responses to other pathogens, CCR2 was important in permitting differentiation of inflammatory monocytes into monocyte-derived DCs that in turn recruited activated CD4^+^ T cells to the lungs during IN LVS infection of mice [19]. However, overall susceptibility to IN LVS infection was not obviously affected. In contrast, in the course of studying the role of MIG/CXCL9 during LVS infections [20], we found that CCR2 KO mice did not survive lower doses of ID LVS infection that were sublethal for wild type mice. We therefore characterized the role of CCR2 during primary responses to systemic vaccination, searched for the CCR2 ligand involved, and determined the overall impact of CCR2 deficiency on secondary protective immunity.

## Materials and methods

### Mice

Male C57BL/6J, B6.129S4-Ccr2^tm1Ifc^/J (CCR2 KO) mice, B6.129S4-Ccl2^tm1Rol^/J (MCP-1/CCL2 KO) mice, and B6.129S4-Ccl7^tm1Ifc^/J (MCP-3/CCL7 KO) mice, ages 6–10 weeks, were purchased from Jackson Laboratories (Bar Harbor, Maine). B6.129S4-Ccl12^tm1Ifc^/AdlJ mice (MCP-5/CCL12 KO) were recovered from cryopreserved sperm by Jackson Laboratories, then shipped to CBER for further in-house breeding. All mice were housed in sterile microisolator cages, and mice were provided with autoclaved food and water *ad libitum*. All animal studies were performed under protocols approved by the Animal Care and Use Committee (ACUC) of CBER/FDA; protocols stressed practices and procedures designed to minimize animal suffering. Within each experiment, all animals were aged-matched.

### Ethics statement

All experiments were performed under protocols approved by the Animal Care and Use committee of CBER (Animal Study Protocols #1993–03 and #1999–25). These protocols meet the standards for humane animal care and use set by the Guide for the Care and Use of Laboratory Animals and PHS policy. Infection studies included frequent observations and observed humane endpoints. At the indicated time points or at the end of a study, animals were euthanized with carbon dioxide inhalation in a euthanasia chamber where carbon dioxide was introduced at the rate of at least 20% of the chamber volume per minute. No animals were subjected to anesthesia. When illness or death was expected due to experimental infections, mice were checked visually by investigators at least twice daily (including weekend days and holidays). Mice that exhibited more than 20% weight loss, or that were unable to ambulate sufficiently to obtain water or food, were humanely sacrificed by carbon dioxide inhalation and scored as deaths.

### Bacteria and growth conditions

*F*. *tularensis* LVS (American Type Culture Collection #29684, Manassas, VA) were grown in modified Mueller-Hinton (MH) broth (Difco Laboratories, Detroit, Michigan) to mid-logarithmic phase as previously described [21,22], then frozen in 0.5 ml aliquots at –80°C. Aliquots were thawed for individual experiments and used immediately. A sample from each batch of bacterial stock used for *in vivo* or *in vitro* studies was subjected to quality control experiments to determine the number of colony forming units (CFU); to determine the proportion of dead bacteria, using the Live/Dead BacLight Bacterial Viability kit (Invitrogen/Life Technologies, Grand Island, NY); to confirm typical bacterial colony morphologies; and to determine the intraperitoneal (IP) and intradermal (ID) LD_50_ and time to death in adult male BALB/cByJ mice [15]. Only bacterial stocks that exhibited an IP LD_50_ of ≤ 3 CFU, time to death between 5–7 days after an IP dose of 10^1^ or 10^2^ CFU, and an ID LD_50_ of ≥ 10^5^ CFU were used for *in vivo* infections.

### Bacterial infections

Groups of C57BL/6J and KO mice were infected ID with doses of LVS ranging from 5 x 100–5 x 10^6^ CFU, delivered in 0.1 ml of sterile phosphate buffered saline (PBS) (Lonza, Walkersville, MD) containing < 0.01 ng of endotoxin/ml. For lethal challenge experiments, mice that survived primary infection and were therefore vaccinated were challenged intraperitoneally (IP) with 1–5 x 10^6^ LVS, as indicated. Each LVS infection dose was plated on MH plates to retrospectively quantitate the actual number of CFU delivered, and colonies were enumerated after 3 days incubation at 37°C/5% CO_2_. Infected mice were monitored and euthanized when unable to reach food and water, according to protocol-established criteria.

### In vivo blocking of CCR2 ligand chemokines

We used MCP-5 KO/CCL12 KO mice treated with anti-CCL2 and anti-CCL7 antibodies to generate “triple chemokine deficient” mice. Chemokines were inhibited *in vivo* by injecting mice with the indicated monoclonal antibodies in a 100 μl volume via IP injection 24 hours prior to LVS infection and again 24 hours after LVS infection, using doses and schedules accordingly to previously published reports. Similar groups were given 100 μl PBS as a control treatment. CCL2 was blocked by administration of 100 μg anti-CCL2 (clone 2H5, BioXCell, West Lebanon, NH) [23]. CCL7 was blocked by administration of 20 μg polyclonal anti-CCL7 (R&D Systems) [24]. Control and treated groups were then administered 10^5^ LVS ID. To confirm depletion of the respective chemokines, one treated mouse and two non-treated mice were euthanized at 48 hours after the second injection of anti-chemokine antibody (*i*.*e*., 72 hours after LVS infection). Lungs, livers, and spleens were then homogenized, and tissue homogenate supernatants were assessed by ELISA for CCL2, CCL7, or CCL12. CCL2 and CCL7 amounts were below the level of detection in the treated mouse in lungs, livers, and spleens.

### Assessment of bacterial organ burdens and tissue pathology

Bacterial burdens in organs were determined by plating at the indicated time points after infection. Mice were euthanized, and organs removed aseptically and transferred to sterile homogenizer bags containing 5 ml of sterile PBS/organ. Organs were disrupted using a Stomacher^®^ (Seward, England), and the homogenates serially diluted and plated onto MH plates for colony enumeration. Organ homogenates were also frozen and stored at -80°C to be used for cytokine analysis. Blood from the femoral artery and heart was also collected and diluted in 0.5 mg/ml EDTA to prevent clotting for subsequent protein analyses, or blood was allowed to clot for sera analyses. For organ homogenates, the limit of detection was 50 CFU/organ. For assessment of antibodies, sera were isolated from whole blood using Sarstedt serum gel microtubes (Fisher Scientific, Pittsburg, PA) and frozen for later analyses.

### Physiological and hematological blood analyses

Blood and serum samples were analyzed by the Department of Laboratory Medicine, Clinical Center, National Institutes of Health in Bethesda, MD. Whole blood complete blood count (CBC) was performed on a Cell Dynn 3700 Analyzer (Abbott Diagnostics, Abbott Park, IL). Serum chemistry analysis was performed on a Siemens Dimension Vista 1500 Analyzer (Siemens Healthcare Diagnostics, Tarrytown, NY).

### Characterization of antibody responses

Mice were individually pre-bled, infected with 5 CFU LVS ID, bled 30 days later, and sera prepared. Titers of specific anti-LVS serum antibodies were determined by ELISA as described previously [25]. Briefly, Immulon 1 plates were coated with live LVS, washed, and blocked with 10% calf serum. Serial dilutions of serum samples were added to coated wells. In each assay, sera from naïve mice was used as a negative control, and sera from LVS-hyperimmune mice, generated by repeated immunization of mice with LVS, was used as a positive control. Horseradish-peroxidase labeled antibodies (anti-IgM or anti-IgG that detects IgG_1_, IgG_2a_, IgG_2b_, and IgG_3_) (Southern Biotech, Birmingham, AL) were added, and ABTS peroxidase substrate (Kirkegaard & Perry Laboratories, Gaithersburg, MD) was used for color development. The end point titer was defined as the lowest dilution of serum that gave an optical density at 405 nm greater than the optical density at 405nm when three standard deviations were added to the OD value of the matched dilution of normal pre-bleed mouse serum, and also greater than 0.025 OD units. Geometric mean titers were calculated from endpoint titer values from 5 or 7 individual mice within a group.

### In vitro overlay co-culture assay

Co-cultures were performed in 24 or 48 well tissue culture plates as described previously [22]. Briefly, bone marrow-derived macrophages (BMMØ) were cultured in complete DMEM (DMEM supplemented with 10% heat-inactivated FCS [HyClone, Logan, UT], 10% L-929-conditioned medium, 0.2 mM L-glutamine, 10 mM HEPES buffer, and 0.1 mM nonessential amino acids) in 24 well plates. After differentiation for 6–7 days, confluent adherent macrophage monolayers were infected for 2 hours with *F*. *tularensis* LVS at a multiplicity of infection (MOI) of 1:20 (bacterium-to-BMMØ), washed, treated for 60 min with 50 μg/ml gentamicin, and washed extensively with antibiotic-free medium. Spleens were aseptically removed from the indicated mice and homogenized in 2% fetal calf serum (FBS) (Hyclone). Single-cell suspensions were generated, red blood cells were lysed using ACK Lysis Buffer, and cell viability was determined using trypan blue dye exclusion. Single-cell suspensions of splenic lymphocytes (5 x 10^6^/well, or as indicated) were added to LVS-infected macrophages. At 72 hours after infection, supernatants from harvested cells were collected and stored at -80°C until analyzed for nitric oxide and cytokines as described below. Intracellular bacterial burdens in adherent infected macrophages were determined by removing supernatants and non-adherent lymphocytes, then lysing the remaining adherent macrophages with water, and plating the resulting lysate. In some experiments, the indicated wells with lymphocytes co-cultured with LVS-infected macrophages were left either untreated, or wells were treated with 25 μg/ml anti-mouse CCR2 antibody (MAB55381, R&D Systems, Minneapolis, MN) or 25 μg/ml of IgG_2b_ isotype control (BD Pharmingen, San Diego, CA), as indicated.

### Flow cytometry

Single cell suspensions prepared from spleens were stained for a panel of murine cell surface markers and analyzed using a Becton-Dickinson LSR II flow cytometer (San Jose, CA) and FlowJo (Tree Star, Inc) software, as previously described [22] with minor modifications. Briefly, cells were washed and resuspended in flow cytometry buffer (PBS/2% serum). Non-specific binding of antibodies was inhibited by blocking Fc receptors with anti-CD16/CD32 (Fc Block; BD Pharmingen) for 10 minutes on ice. To discriminate live from dead cells, a staining step was performed using a commercially available kit and following the manufacturer’s instructions (Live/dead Staining Kit, Invitrogen). The cells were then washed and stained for cell surface markers. Antibody concentrations were optimized separately for use in multiparameter staining protocols, using appropriate fluorochrome-labeled isotype matched control antibodies. The following antibodies were used: anti-B220 (clone RA3-6B2), anti-CD19 (clone 1D3), anti-TCRβ (clone H57-597), anti-CD11b (clone M1/70), anti-Ly6C (clone AL-21), anti-CD11c (clone HL3), and anti-CCR2 (clone 475301), each labeled with a variety of fluorochromes as needed (above antibodies were purchased from BD Pharmingen or R&D Systems). Representative dot plots to illustrate gating and analyses strategies are provided in S1 File.

### Measurement of cytokines and nitrite in tissue culture supernatants

Sera and supernatants recovered from *in vitro* co-cultures were assayed for mediators of interest using standard sandwich ELISAs, according to the manufacturer’s instructions (BD Pharmingen; Cayman Biochemical, Ann Arbor, MI; and Life Diagnostics, West Chester, PA). The absorbance was read at 405 nm on a VersaMax tunable microplate reader with a reference wavelength of 630 nm (Molecular Devices, Sunnyvale, CA). Cytokine concentrations were determined by comparing unknown values to standard curves made with recombinant proteins at known concentrations (BD Pharmingen; Life Diagnostics; Cayman Chemical), using four-parameter fit regression in the SOFTmax Pro ELISA analysis software (Molecular Devices). Nitric oxide was estimated in culture supernatants using the Griess reaction and a commercial Griess reagent (Life Technologies, Grand Island, NY); absorbance was measured at 548 nm, and nitrite (NO_2_) was measured by comparison to serially diluted NaNO_2_ as a standard, using four-parameter fit regression as described above.

### Quantitation of MCP-1 and MCP-5 in organ homogenates using Quantibody Cytokine Array

During initial screening studies, a panel of murine cytokines and chemokines in spleen homogenates were assessed using Quantibody Cytokine Arrays 4, 5, and 6 (Ray Biotech, Norcross, GA), according to the manufacturer’s protocols. Proteins including MCP-1/CCL2 and MCP-5/CCL12 (shown here) were quantitated using standard curves of serial dilutions of a known quantity of each protein run in tandem on each chip.

### Statistical analyses

The significance of differences between the indicated parameters was assessed using Student’s *t* test or ANOVA (SigmaPlot, Systat Software, Inc., San Jose, CA), and p < 0.05 taken as an indicator of significant differences. Differences in survival were assessed using log-rank (Mantel-Cox) tests (GraphPad Prism, San Diego, CA).

## Results

### Characterization of the role of CCR2 during primary ID LVS infection

Previous studies demonstrated that splenocytes from mice infected with 10^5^ LVS ID produced MCP-1, considered to be a major ligand for CCR2 *in vivo* [26]. Further, recruitment and development of CCR2^+^ lung inflammatory monocytes was impaired in mice administered a sublethal dose of 2 x 10^2^ LVS IN in the absence of CCR2 [19]. Here, we directly evaluated systemic inflammatory monocytes during parenteral ID LVS infection of wild type mice or mice deficient in CCR2. Wild type C57BL/6J or CCR2 KO mice were infected with 10^4^ LVS ID, and two days later spleens and bone marrow were evaluated for numbers of lymphoid and myeloid cell subpopulations. Compared to LVS-infected wild type mice, the proportions of total myeloid cells were similar in spleens but increased in bone marrow of LVS-infected CCR2 KO mice (Table 1). CCR2^+^ cells were not detectable in either tissue in CCR2 KO mice, confirming the expected phenotype. Most notably, spleens from LVS-infected CCR2 KO mice contained considerably fewer CD11b^+^Ly6C^+^ inflammatory monocytes than those from wild type mice. Clinical evaluation of complete blood counts (CBC) suggested that the blood of infected CCR2 KO mice occasionally had reduced numbers of both monocytes and lymphocytes, but levels were variable (S2 File).

**Table 1:** Splenocyte and bone marrow myeloid cell subpopulations in mice infected with 10^4^ LVS ID.

	Percent of cells with the indicated markers					
Cell type and markers		Spleen			Bone marrow	
	WT, PBS	WT, LVS	CCR2 KO, LVS	WT, PBS	WT, LVS	CCR2 KO, LVS
Total myeloid (CD19^-^ B220^-^ TCRβ^-^)	5.8±2.6	13.6±4.2^a^	12.5±9.2^a^	65.8	60.4±11.4^d^	71.6±6.6^d^
Monocytes (CD11b^+^Ly6C^+^)	17.9±4.1	37.2±16.0^b^	22.6±14.4^b^	79.3	74.5±3.7^e^	78.3±3.3^e^
CCR2^+^ myeloid (CD11b^+^Ly6C^+^CCR2^+^)	28.9±3.9	21.3±8.7^c^	3.4±1.1*^c^	13.0	4.7±0.9^f^	1.9±0.4*^f^

Wild type C57BL/6J and CCR2 KO mice were treated with PBS or infected ID with 10^4^ CFU LVS. Groups of three mice from each group were sacrificed on day 2 after infection; spleens and bone marrow were harvested, and single cell suspensions were prepared for analyses by multi-parameter flow cytometry. Cells were analyzed on an LSR II cytometer using appropriate single-color compensation and isotype controls; only CD45^+^, live non-aggregated cells were included in analyses.

*, CCR2^+^ cells were below the limit of detection; values entered were considered background levels but are shown for comparison to the values found for cells from wild type mice using the gating strategy applied. Data represent the mean ± standard error of percent total live CD45^+^ cells from four representative experiments, each with three mice per group, that assessed major leukocyte subpopulations and one that included assessment of CCR2 expression. Proportions of cells in PBS-treated (uninfected) wild type C57BL/6J mice are shown for reference. P values of comparisons between LVS-infected WT vs. KO values are:

^a^*p* = 0.3910;

^b^*p* = 0.0148;

^c^*p* = 0.0341;

^d^*p* = 0.0046;

^e^*p* = 0.0116;

^f^*p* = 0.0171.

During LVS infection, MCP/CCL proteins that are ligands for CCR2 in mice are found systemically [26,27]. To further confirm that representative CCL/MCP proteins were produced following infection, C57BL/6J or CCR2 KO mice were infected ID with 10^4^ CFU of LVS. At days 2 and 6 after infection, mice were euthanized, and spleen homogenates were assessed for MCP proteins. Both MCP-1/CCL2 and MCP-5/CCL12 were readily detectable in spleens from both mouse strains on day 2, and amounts increased by day 6 (S3 File); amounts were variable between mice, but levels of both chemokines were generally higher in CCR2 KO homogenates on day 6 compared to those from WT mice, possibly due to the lack of chemokine binding to CCR2.

**Fig 1:**
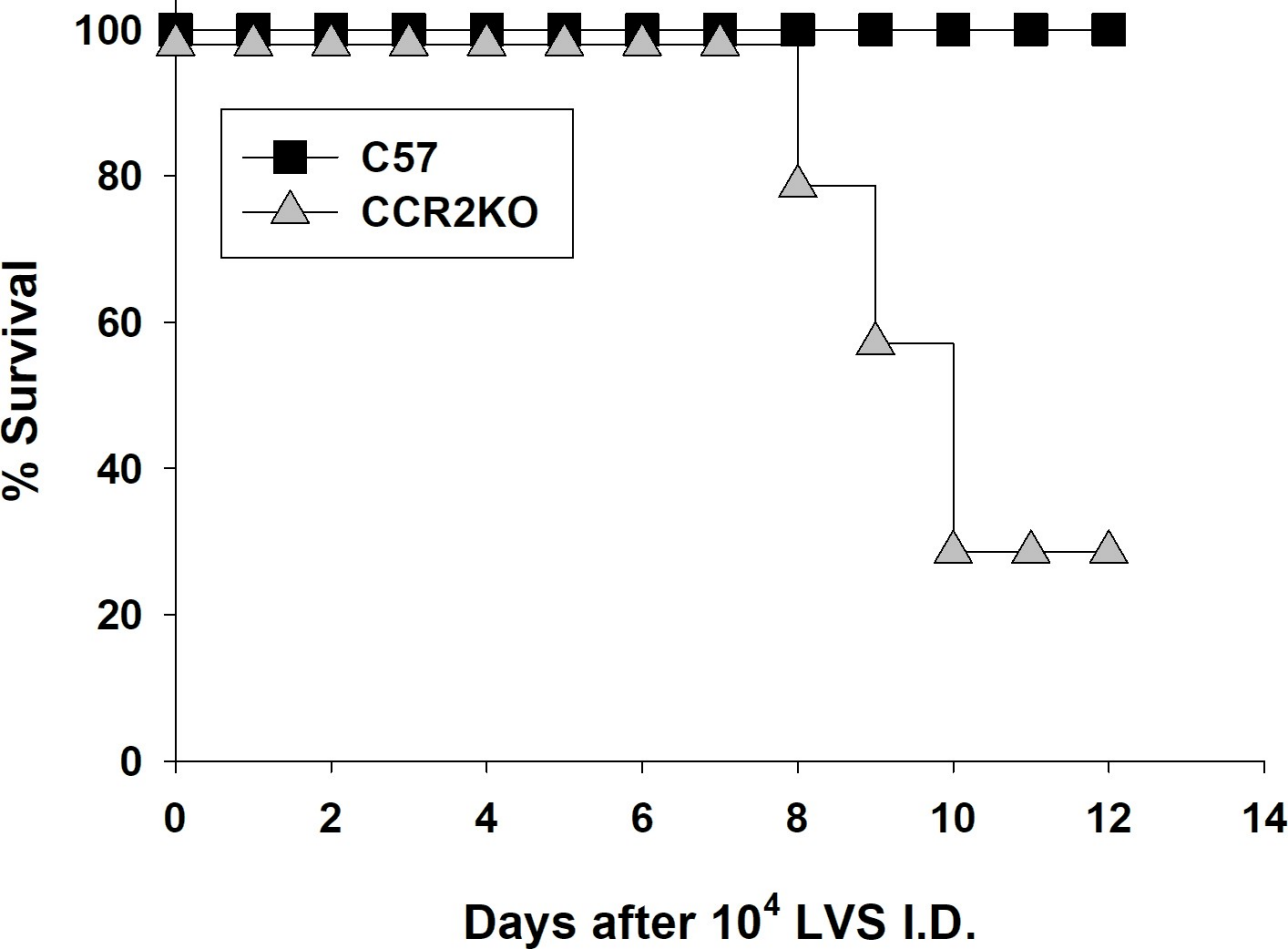
CCR2 KO mice exhibit increased susceptibility to intradermal *Francisella tularensis* LVS infection compared to C57BL/6J mice. Groups of three to five C57BL/6J or CCR2 KO mice were infected ID with a target dose of 10^4^ LVS; actual doses were confirmed by retrospective plate count and were within 15% of the target. Survival was monitored for at least 30 days, but no further deaths occurred after day 10. Results are combined from three independent experiments of similar design and outcome that used a total of 15 C57BL/6J mice (black squares) and 14 CCR2 KO mice (gray triangles). Overlapping lines are offset for clarity. The survival of LVS-infected C57BL/6J mice was significantly different from that of LVS-infected CCR2 KO mice, with p < 0.0001 per a log-rank (Mantel-Cox) test.

**Fig 2:**
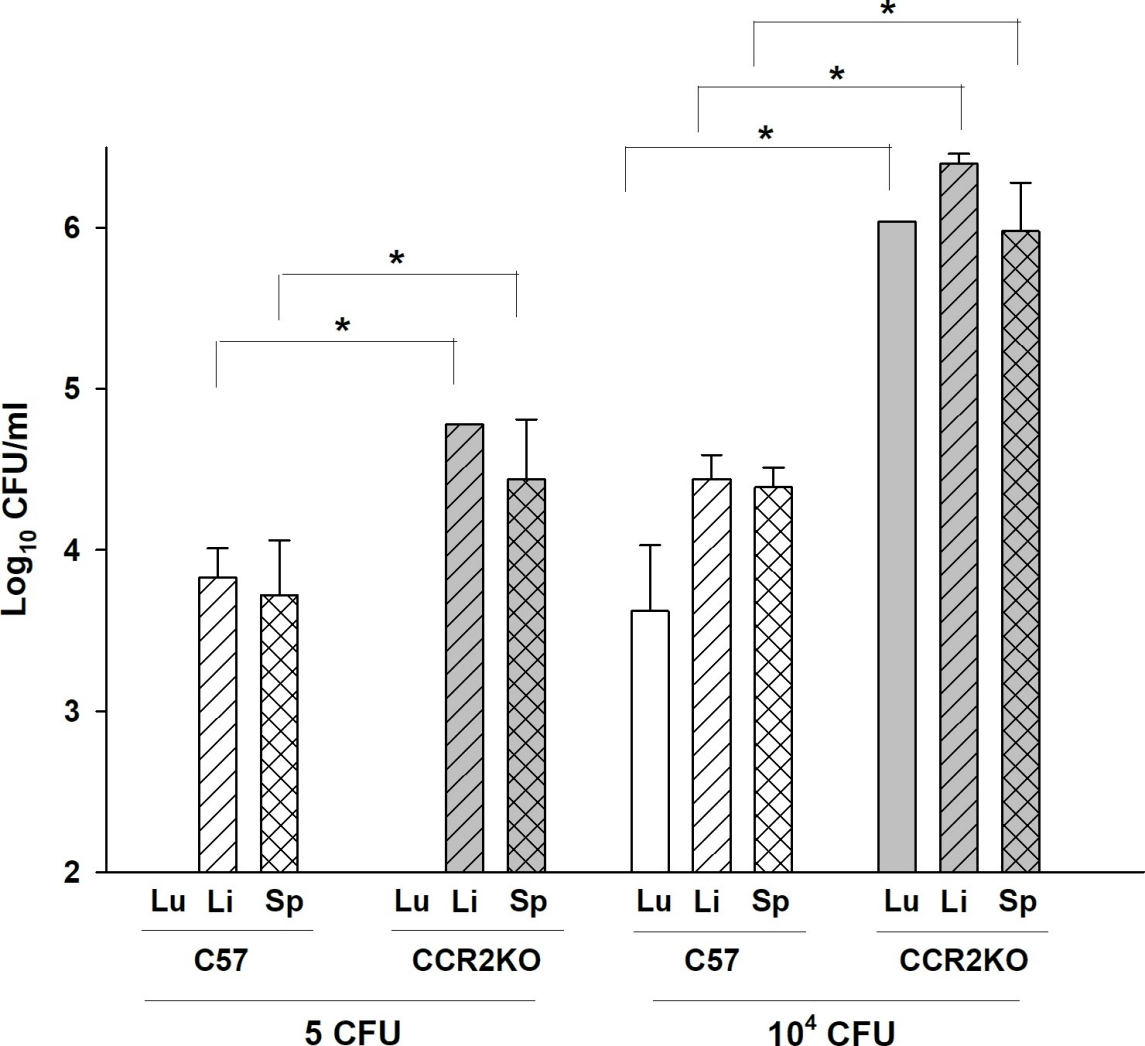
CCR2 KO mice exhibit increased bacterial burdens in organs following ID LVS infection with 10^4^ LVS. C57BL/6J or CCR2 KO mice were infected ID with 5 or 10^4^ CFU of LVS. Three mice per group were sacrificed at day 6 after infection, and lungs (open bars), livers (hatched bars), and spleens (cross hatched bars) were harvested, homogenized, and plated on MH plates to enumerate CFU. Each bar represents the average ± standard deviation of three samples from the indicated mice using the stated dose. Results are shown from one representative experiment of three of similar design and outcome. * p ≤ 0.05 by Student’s *t* test between the bracketed comparisons between each type of organ at each dose.

**Fig 3:**
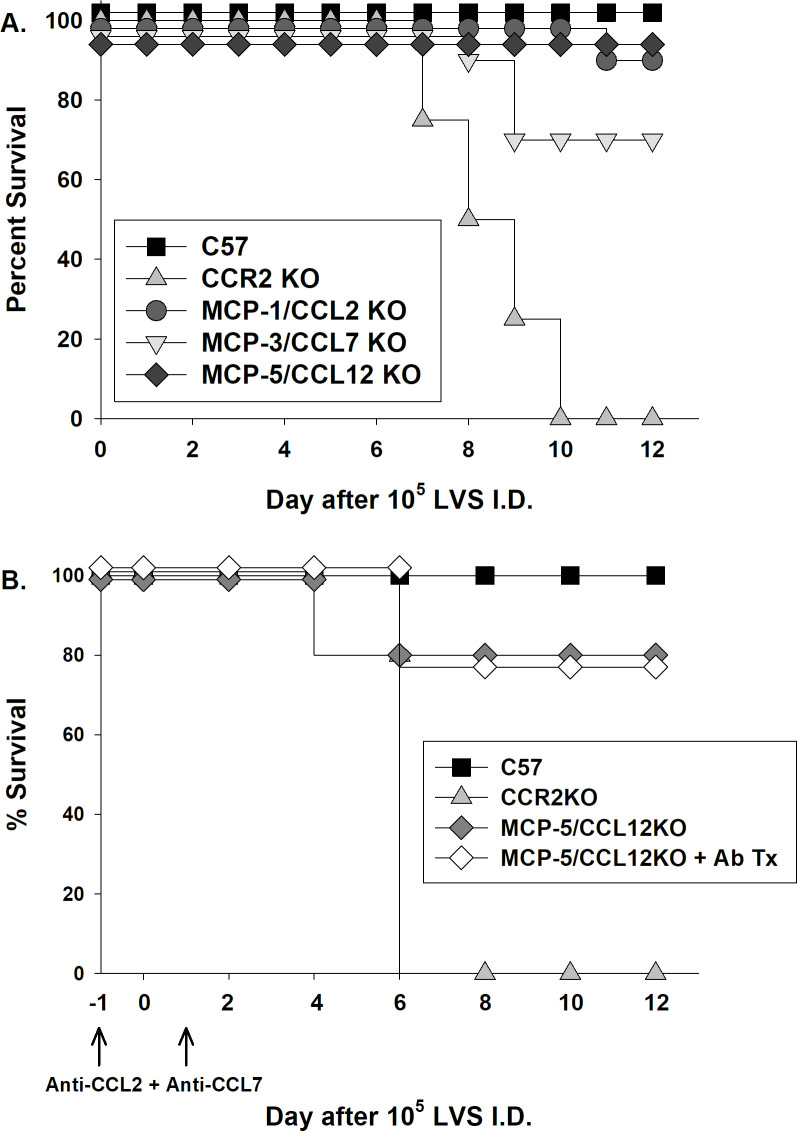
The increased susceptibility of CCR2 KO mice to intradermal *Francisella tularensis* LVS is not explained by the activities of the major CCR2 ligands MCP-1/CCL2, MCP-3/CCL7, and MCP-5/CCL12. A) Groups of four to five C57BL/6J, CCR2 KO, MCP-1/CCL2 KO, MCP-3/CCL7 KO, and MCP-5/CCL12 KO mice were infected ID with a target dose of 10^5^ LVS; actual doses were confirmed by retrospective plate count and were within 15% of the target. Survival was monitored for at least 30 days, but no further deaths occurred after day 10. Results are combined from up to three independent experiments of similar design and outcome that targeted this dose and used a total of 20 C57BL/6J mice, 4 CCR2 KO mice, 8 MCP-1/CCL2 KO, 10 MCP-3/CCL7 KO, and 10 MCP-5/CCL12 KO mice. Overlapping lines are offset for clarity. The survival of LVS-infected CCR2 KO mice was significantly different from that of LVS-infected MCP-1/CCL2, MCP-3/CCL7, or MCP-5/CCL12 KO mice, with p < 0.0001 for all pairwise comparisons per log-rank (Mantel-Cox) tests. B) MCP-5/CCL12 KO mice were treated with anti-CCL2 and anti-CCL7 as described in Methods. One day later, groups of five C57BL/6J, CCR2 KO, MCP-5/CCL12 KO, and antibody-treated MCP-5/CCL12 KO mice were infected ID with a target dose of 10^5^ LVS; actual doses were confirmed by retrospective plate count and were within 15% of the target. One day after infection, MCP-5/CCL12 KO mice were treated again with anti-CCL2 and anti-CCL7 to maintain depletion. Survival was monitored for at least 30 days, but no further deaths occurred after day 6. Results are shown from one representative experiment of two independent experiments of similar design and outcome. Overlapping lines are offset for clarity. The survival of LVS-infected CCR2 KO mice was significantly different from that of LVS-infected MCP-5/CCL12 KO mice (p = 0.04) or antibody-treated MCP-5/CCL12 KO mice (p = 0.01), per log-rank (Mantel-Cox) tests.

The LVS ID LD_50_ in C57BL/6J mice is over 10^6^ CFU; most mice readily survive, and are typically vaccinated by, doses up to 5 x 10^5^ LVS ID [14,15]. Initial experiments suggested that CCR2 KO mice became moribund following administration of considerably lower doses of LVS ID. We therefore evaluated the susceptibility of CCR2 KO mice in further detail by comparing outcomes following administration of LVS doses ranging from 5 x 10^0^ to 10^6^ CFU ID. CCR2 KO mice succumbed to infection with doses as low as 10^2^ LVS ID (S4 File), and 70% of CCR2 KO mice given a dose of 10^4^ LVS ID died between 8–10 days after infection (Fig 1). Consistent with previous results, all wild type mice survived these doses and did not exhibit any visible symptoms during infection. Increasing LVS doses resulted in more rapid times to death of infected CCR2 KO mice, which ranged between 5 and 11 days after infection (S4 File; see Fig 3). Further, morbidity was accompanied by high organ burdens. By day 6 after infection, CCR2 KO mice given a dose of 10^4^ LVS ID had ~ 100-fold more bacteria in spleens, livers, and lungs than the corresponding C57BL/6J mice (Fig 2). Even infection with a sublethal 5 CFU dose of LVS ID resulted in ~ 5-fold more bacteria in spleen and livers of CCR2 KO mice by day 6. Of note, bacterial burdens were not detectable in lungs of either type of mice given this low dose (Fig 2), suggesting similar patterns of bacterial dissemination. Clinical evaluation of blood chemistry indicated that the blood of CCR2 KO mice infected with 10^4^ LVS ID had increased levels of AST and ALT compared to wild type mice (S2 File), suggesting liver dysfunction accompanied high bacterial burdens. Therefore, CCR2 mice are severely susceptible to systemic ID LVS infection.

### Evaluation of individual CCR2 chemokine ligands operative during lethal primary ID LVS infection

To determine the chemokines involved in the susceptibility of CCR2 KO mice, we evaluated the roles of individual CCR2 ligand chemokines during ID LVS infection. In contrast to CCR2 KO mice, MCP-1/CCL2 KO mice, MCP-3/CCL7 KO mice, and MCP-5/CCL12 KO mice largely survived doses of 10^5^ LVS ID without developing symptoms (Fig 3A), suggesting that no single chemokine ligand’s activities explained the phenotype of CCR2 KO mice. We therefore evaluated the possibility that multiple ligands, acting cooperatively, were involved. We treated MCP-5/CCL12 KO mice *in vivo* with antibodies to block both MCP-1/CCL2 and MCP-3/CCL7, resulting in “triple chemokine deficient” mice [23,24]. Treated mice were then infected with a dose of 10^5^ LVS ID. Neutralization of chemokines was effective, as each respective chemokine could no longer be detected in spleens of LVS-infected, antibody-treated mice (see Materials and Methods). Nonetheless, at this LVS infection dose, mice lacking circulating MCP-1/CCL2, MCP-3/CCL7, and MCP-5/CCL12 largely survived, while CCR2 KO mice did not (Fig 3B). Therefore, the profound susceptibility of CCR2 KO mice cannot be directly attributed to activities of this combination of chemokines, which are considered to be the major ligands for CCR2 in mice.

### Determination of the role of CCR2 in vaccination against secondary lethal *Francisella* challenge

The relatively long time to death of CCR2 mice given lower doses of LVS ID (Figs 1 and 3 and S4 File) raised the possibility that, in addition to CCR2’s role during innate immune responses, susceptibility was also related to poor development of T cell-mediated adaptive immune responses; T cell activities are necessary to clear primary LVS ID infections and establish protection against secondary challenge [14,15]. We therefore next evaluated whether CCR2 KO mice that survived a low 5 CFU dose of LVS ID, and thus were effectively vaccinated [14,15], generated appropriate adaptive memory responses to *Francisella*. CCR2 KO and wild type C57BL/6J mice were pre-bled and then administered 5 CFU LVS ID. One month later, sera were obtained from individual mice and evaluated for binding to whole LVS bacteria. No anti-LVS antibodies were detected in pre-immunization serum samples (limit of detection, 1:20 dilution of serum), and all individual mice exhibited readily detectable anti-LVS antibodies after immunization; this confirmed that the low numbers of LVS bacteria administered (a target dose of 5 CFU) were delivered appropriately. In two experiments using groups of five or seven mice, the combined geometric mean endpoint titers of IgM anti-LVS antibodies in LVS-infected C57BL/6J mice on day 30 were 2.51 ± 0.35 and 2.42 ± 0.23 in LVS-infected CCR2 KO mice, which were comparable (p = 0.30). Geometric mean titers of IgG anti-LVS antibodies of 3.97 ± 0.27 were higher in CCR2 KO mice compared to those in C57Bl/6J mice, which exhibited titers 3.49 ± 0.33 (p = 0.007). This increase may reflect increased bacterial burdens in CCR2 KO mice (Fig 2).

**Fig 4:**
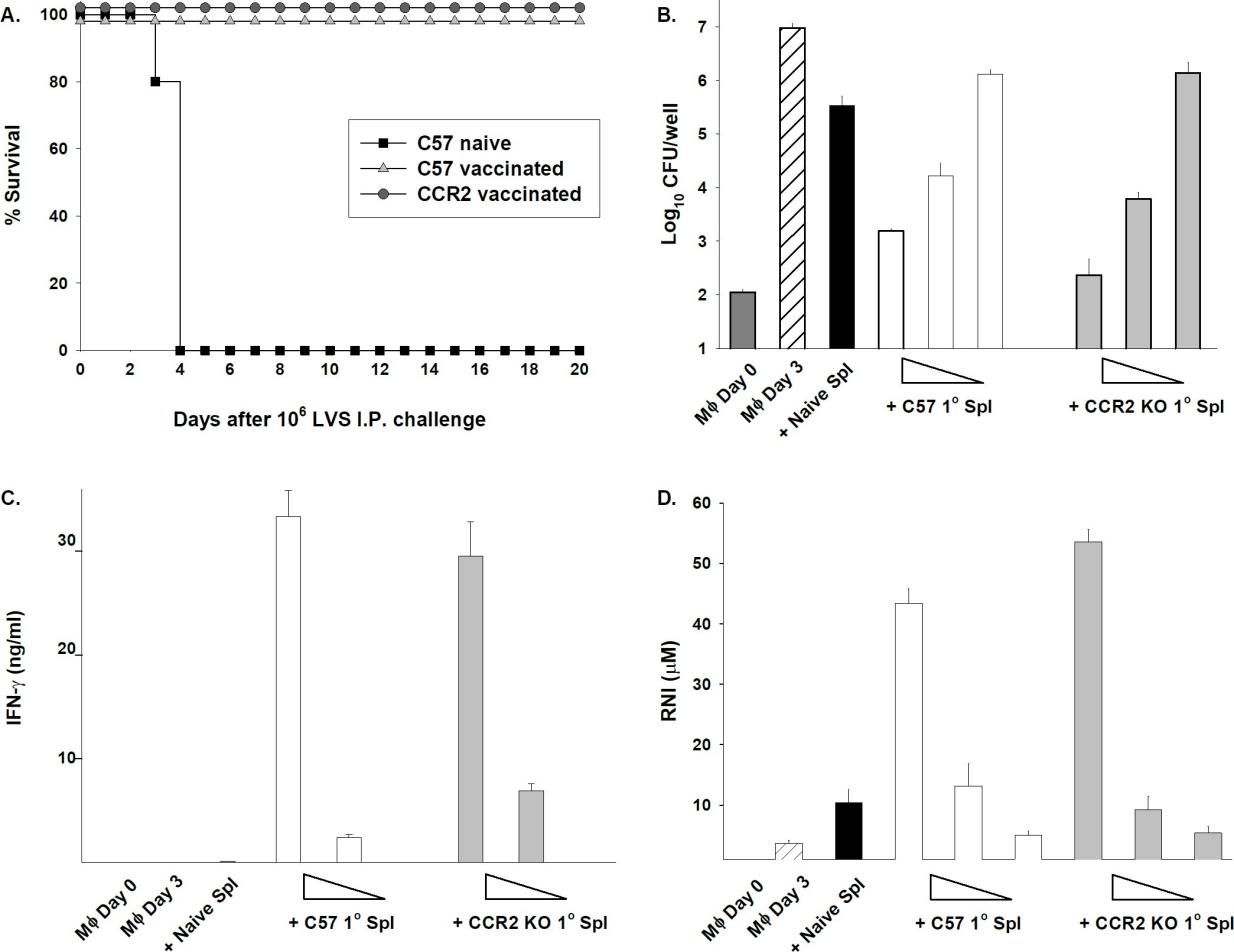
LVS-immune CCR2 KO mice are similar to C57BL/6J mice in surviving maximal secondary lethal LVS challenge and generating immune T cells that control intramacrophage LVS growth. A) Groups of five C57BL/6J or CCR2 KO mice were treated with PBS (naïve) or vaccinated by ID infection with 5 x 10^0^ CFU LVS. After one month, all mice were challenged with 10^6^ LVS IP. Actual doses were confirmed by retrospective plate count and were within 15% of the target. Survival was monitored for at least 30 days, but no further deaths occurred after day 6. Results are shown from one representative experiment of three independent experiments of similar design and outcome that used a total of 15 C57BL/6J mice and 14 CCR2 KO mice. Overlapping lines are offset for clarity. The survival of LVS-challenged naïve C57BL/6J mice was significantly different from that of LVS-challenged primed C57BL/6J or primed CCR2 KO mice, with p = 0.0035 for both pairwise comparisons per log-rank (Mantel-Cox) tests. B) Splenocytes from the same LVS-immune C57BL/6J and CCR2 KO mice were isolated and pooled (designated as “1° cells” on the graphs); splenocytes were also obtained from naive mice. Bone marrow-derived macrophages (Mϕ) from C57BL/6J mice were infected with LVS, and then splenocyte populations were added to triplicate wells of infected macrophages. For naïve and the first series of LVS-immune splenocytes, 5 x 10^6^ lymphocytes were added per well to triplicate wells per condition. For titration of lymphocytes, 2.5 x 10^6^ or 1 x 10^6^ immune splenocytes were added, depicted as to represent decreasing numbers of added lymphocytes. Intracellular bacterial burdens represented as CFU/well were assessed immediately after infection at 0 hours (designated as Mϕ Day 0 on the graphs) and at 72 hours (Mϕ Day 3) after the initiation of co-cultures and compared to numbers of recovered CFU/well from infected macrophages co-cultured with added lymphocytes, as indicated. C) Supernatants were harvested from the co-cultures shown in B and assayed for amounts of IFN-γ by ELISA. Results shown are the mean of triplicate wells. C) Supernatants were harvested from the co-cultures shown in B and assayed for assayed for RNI levels using the Griess reagent. Results shown are the mean of triplicate wells. Results shown in B, C, and D are from one representative experiment of seven of similar design and outcome.

LVS-vaccinated mice were then challenged *in vivo* with a maximal lethal dose of LVS IP [14,15]. In parallel, the remaining mice from the same vaccination group were euthanized (without challenge) to obtain splenocytes for *in vitro* co-culture assays. Naïve mice that were challenged with 10^6^ LVS IP rapidly succumbed, but most C57BL/6J wild type mice and all CCR2 KO mice survived (Fig 4A). Addition of naïve splenocytes to LVS-infected bone marrow-derived macrophages had little impact on the intramacrophage replication of LVS, but splenocytes from LVS-immune wild type C57BL/6J mice and CCR2 KO mice both controlled intramacrophage bacterial growth (Fig 4B). Moreover, titration of numbers of added splenocytes to LVS-infected macrophages indicated that the frequency of LVS-immune effector cells, and thus the potency of control, was comparable between C57BL/6J and CCR2 KO splenocytes on a *per cell* basis (Fig 4B). Further, amounts of the important mediators IFN-γ (Fig 4C) and nitric oxide (Fig 4D) produced in co-cultures with each type of LVS-immune splenocytes were also comparable. Therefore, although CCR2 KO mice are exquisitely susceptible to primary ID LVS infection, once vaccinated by primary LVS infection, normal and protective adaptive immune memory responses can be generated in the absence of CCR2.

## Discussion

Like other intracellular pathogens, previous studies suggested that CCR2-related chemokines were produced during *Francisella* infections and played important roles in host responses. Here, we demonstrated directly that CCR2 deficiency results in substantially increased susceptibility to systemic infection with an attenuated strain of *F*. *tularensis*, leading to poor control of bacterial burdens (Fig 2) and death within 1–2 weeks (Fig 1). Surprisingly, the chemokines responsible for susceptibility could not be identified either individually or in combination: mice deficient in MCP-1/CCL2, MCP-3/CCL7 KO mice, or MCP-5/CCL12, the major known murine ligands for CCR2, were not unusually susceptible following ID LVS infection (Fig 3). Numbers and proportions of splenic monocytes were modestly reduced in tissues of LVS-infected CCR2 KO mice compared to those in LVS-infected wild type mice, but no other evidence pointed toward a gross innate immune response deficiency (Table 1). The time course of development of morbidity of CCR2 KO mice infected with LVS ID raised the possibility that defects were related to adaptive immune responses. However, CCR2 KO that were vaccinated by inoculation with low doses of LVS ID produced high levels of serum *Francisella*-specific antibodies, developed abundant immune lymphocytes that controlled intramacrophage *Francisella* growth to the same degree as lymphocytes from wild type mice, and survived maximal doses of secondary lethal *Francisella* LVS challenge IP (Fig 4). Therefore, despite deficiencies in inflammatory monocytes, optimal protective immunity to *Francisella* can readily develop in the absence of CCR2.

This is the first report of morbidity of CCR2 KO mice in any *Francisella* infection. Other studies used a relatively low dose of 2 x 10^2^ LVS administered intranasally (IN), which apparently was not lethal for CCR2 KO mice [19]; morbidity was not explicitly discussed, but the IN LD_50_ of LVS in C57BL/6J mice is typically about 10^3^–5 x 10^3^ CFU, and therefore those data imply that the susceptibility of CCR2 KO mice to IN LVS infection is not different from wild type mice. Another study compared responses of C57BL/6J and CCR2 KO mice to an IV dose of 2 x 10^4^ LVS, but most studies were performed using mice infected for only 24 hours [18], well before morbidity develops from LVS infection. Route-dependent differences in the susceptibility of CCR2-deficient mice have been noted previously during primary infection with other intracellular bacteria. For example, CCR2 KO mice were considerably more susceptible to *M*. *tuberculosis* administered IV [11], but not to *M*. *tuberculosis* administered by aerosol. The latter studies demonstrated that bacterial burdens were similar in wild type and CCR2 KO mice, despite exhibiting reduced migration of macrophages into infected lungs [12], which is similar to outcomes in CCR2 KO mice infected with LVS IN [19]. Collectively, the results imply that reduced migration of inflammatory monocytes in the absence of CCR2, and any resulting reductions in cells that differentiate from these, has less impact on survival of respiratory infections than on systemic infections.

A number of reports have documented production of the known CCR2 ligands in *Francisella*-infected mice and from murine and human cells stimulated by *Francisella*. Following stimulation with *Francisella*, MCP-1/CCL2 is made *in vitro* by mouse peritoneal cells [28], human peripheral blood mononuclear cells [29], human umbilical vein endothelial cells [30], human monocyte-derived macrophages [31], and human monocytes [32]. Mice given LVS IN have readily detectable MCP-1/CCL2 *in vivo* in lung homogenates within a week after infection [33,34], and splenocytes from mice infected with LVS ID produce MCP-1 *ex vivo* [26]. Here, we confirmed that spleens of both wild type and CCR2 KO mice contained MCP-1/CCL2 and MCP-5/CCL12 within two days of LVS infection; reagents to quantitate MCP3 were not available. We focused on MCP-1/CCL2 because most researchers have considered MCP-1/CCL2 to be the major *in vivo* ligand for CCR2 [2,3,5].

Surprisingly, mice that were deficient in MCP-1/CCL2, MCP-3/CCL7, and MCP-5/CCL12, individually or collectively, were not nearly as susceptible to ID LVS infection as CCR2 KO mice (Fig 3). Although MCP-2/CCL8 is a CCR2 ligand in human cells, in mice this chemokine only binds CCR8 and not CCR2 [35], and thus CCL8/MCP-8 is not a candidate. We cannot rule out the possibility that insufficient antibody neutralization, or insufficient antibody localization, in “triple chemokine deficient” mice fails to reveal cooperativity or redundancy between these three cytokines that would explain the morbidity of CCR2 KO mice following ID LVS infection. However, we think this explanation is unlikely. Instead, we favor the interpretation that the results imply the existence of another novel murine CCR2 ligand that awaits future discovery.

It is also possible that an excess of circulating ligands that results from the complete absence of CCR2 is pathologic and contributes to morbidity, as was suggested in studies of chemokine receptor redundancy [4]. This notion may be consistent with the results of one study, in which BALB/c mice were administered dose of LVS IV that approximate the LD_50_ dose, then mice that survived were compared to those that became moribund. Spleens of moribund mice contained nanogram amounts of CCL2/MCP-1, while levels in surviving mice were below the limit of detection. CCL2/MCP-2 was therefore considered a marker of mortality, along with IL-6 and MIP2 [36]. Nonetheless, the substantial susceptibility of CCR2 KO mice to ID LVS infection seems unlikely to be completely explained by chemokine excess. Instead, the most likely explanation relates to impaired trafficking of CCR2^+^ inflammatory monocytes during primary responses to LVS.

A variety of studies indicate that CCR2^+^ inflammatory monocytes migrate from bone marrow through blood to infectious foci, then differentiate into monocyte-derived DCs within tissues; these cells are typically Ly6C^hi^ CD11b^hi^ MHCII^+^ CD11c^int^ [11,37,38]. However, we found relatively minor and variable reductions in monocytes in spleens of CCR2 KO mice given LVS ID (Table 1). In IV *Listeria* infections, a subset of monocyte-derived DCs in spleens dubbed TIP-DCs contribute to bacterial control by producing TNF-α and inducible nitric oxide synthase–producing DCs [9,10]. Here, however, in two experiments using three mice per group, spleens of CCR2 KO mice infected with 10^2^ LVS ID exhibited 2.5% ± 2.8 TNF^+^ CD11c^+^ cells on day 6, while C57BL/6J mice had 5.9% ± 2.4% TNF^+^ CD11c^+^ cells, as determined by intracellular cytokine staining. This suggests that CCR2-dependent TIP-DCs are not a major feature of systemic *Francisella* infection.

The time course of morbidity in LVS-infected CCR2 KO mice reflected the time course of development of adaptive immune responses to LVS, which begins to develop about 7–10 days after LVS vaccination [14,15]. The idea of CCR2 deficiency leading to adaptive immunity defects is consistent with evidence that CCR2-dependent monocyte-derived DCs are important during priming of adaptive CD4^+^ T cell responses. This interaction plays a role in murine responses to several pathogens for which Th1 T cells are critical, including *M*. *tuberculosis* [11], the parasite *Leishmania major* [39], and fungi such as *Histoplasma capsulatum* [40]. Moreover, MCP-1/CCL2 found in blood and tissue homogenates was identified as a potential correlate of protection in mice vaccinated with live attenuated *Francisella* mutants and challenged with fully virulent *Francisella* [27]. We therefore carefully considered the possibility that CCR2 deficiency resulted in defects in adaptive T cell and/or B cell responses. However, we found no evidence for this hypothesis. Instead, CCR2 KO mice given a sublethal low dose ID LVS infection produced abundant and indeed increased serum anti-*Francisella* IgG antibodies, suggesting good development of helper T cells, and readily survived the largest available lethal LVS challenge dose as well as wild type mice (Fig 4A). Further, LVS-immune splenocytes from vaccinated CCR2 KO were comparable in frequency and potency to those from wild type mice in their capacity to control intramacrophage LVS replication, and to produce IFN-γ and NO (Fig 4B–4D). We have previously shown that only immune CD4^+^ and CD8^+^ effector T cells perform these functions in this *in vitro* co-culture setting [22]. Thus, by the important functional criteria of control of intramacrophage bacterial replication *in vitro* and survival of secondary challenge *in vivo*, LVS-immune CCR2 KO mice have no obvious defect in T cell priming, effector functions, or memory.

Although previous studies have characterized the course of a number of primary infections in CCR2 KO mice (see above), to our knowledge this is the first report that tests the importance of CCR2 in the context of vaccination and secondary challenge. The results clearly demonstrate that CCR2-dependent functions are dispensable for development of maximal protective immunity against this model intracellular bacterium, and therefore support targeting other pathways during the search for improved vaccination strategies against this large and important class of pathogens.

## Supporting information

S1 File Fig flow gating. (PDF)

S2 File Clinical chemistry and hematology. (XLSX)

S3 File MCP1 MCP5 quantitation in spleen homogenates. (XLSX)

S4 File Fig CCR2 infection varied doses. (PDF)
