# Supplementary material for: Deficiency in CCR2 increases susceptibility of mice to infection with an intracellular pathogen, Francisella tularensis LVS, but does not impair development of protective immunity
Source: PLoS One. 2021 Mar 24;16(3):e0249142. doi: 10.1371/journal.pone.0249142 (PMC7990183; doi:10.1371/journal.pone.0249142)
Supplement: S1 File — (PDF) [file pone.0249142.s001.pdf]

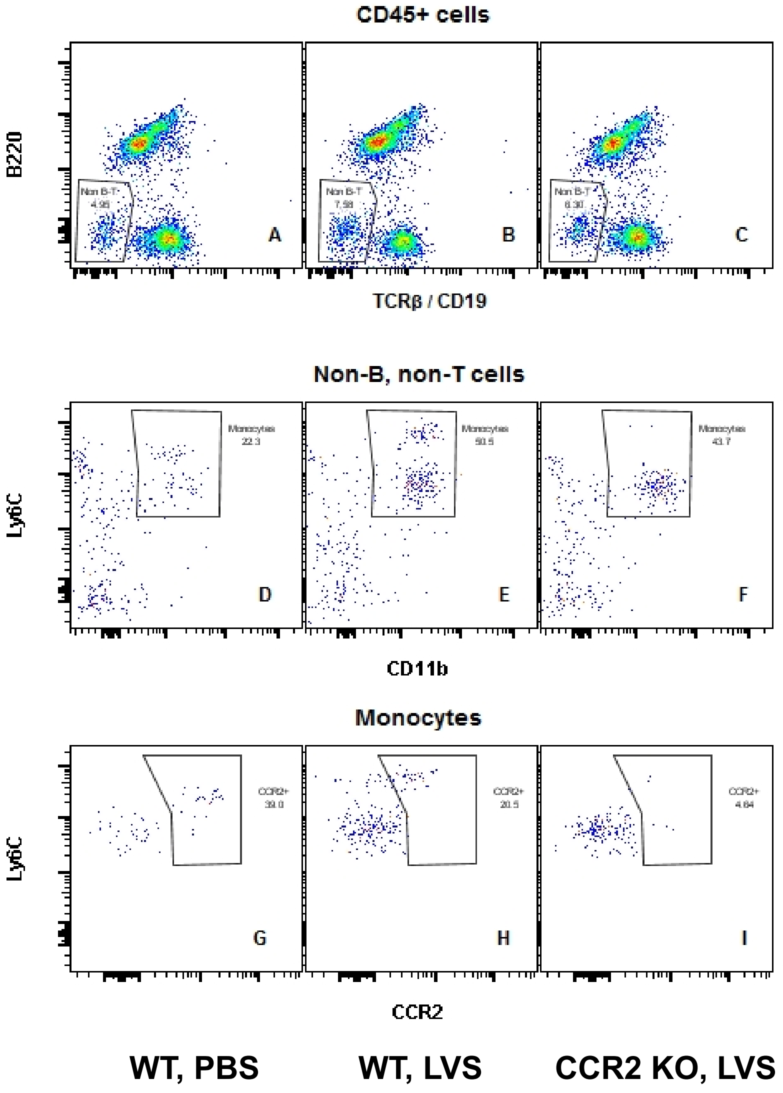

S1 File Figure. Gating strategy to evaluate myeloid cells and CCR2-expressing monocytes. Splenocytes from naïve C57BL/6 (panels A, D and G), LVS-infected C57BL/6 (panels B, E and H) and LVS-infected CCR2 KO (panels C, F and I) mice were analyzed by flow cytometry. After exclusion of fragments, aggregates and dead cells, $CD45^+$ cells were further gated to exclude B and T cells (panels A, B and C). The remaining non-B, non-T cells were gated for $CD11b^+$ $Ly6C^+$ cells, which represent monocytes (panels D, E and F). Monocytes were then further gated for $CCR2^+$ cells. Shown is a representative gating strategy from one animal for each group. A similar gating strategy was used to analyze bone marrow cells.
