# Supplementary material for: Deficiency in CCR2 increases susceptibility of mice to infection with an intracellular pathogen, Francisella tularensis LVS, but does not impair development of protective immunity
Source: PLoS One. 2021 Mar 24;16(3):e0249142. doi: 10.1371/journal.pone.0249142 (PMC7990183; doi:10.1371/journal.pone.0249142)
Supplement: S4 File — (PDF) [file pone.0249142.s004.pdf]

S4 File Fig.

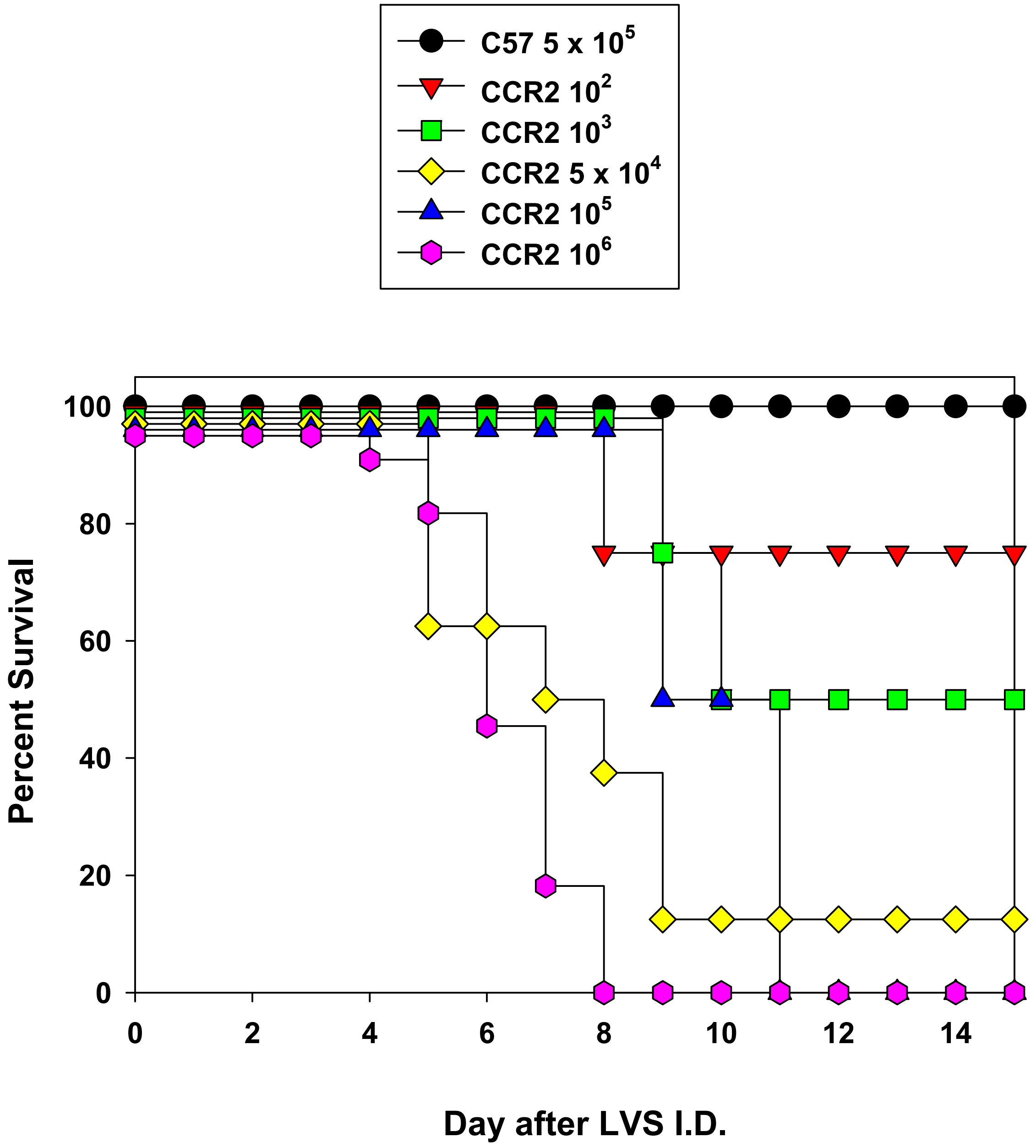

S4 Fig. CCR2 KO mice exhibit increased susceptibility to intradermal *Francisella tularensis* LVS infection compared to C57BL/6J mice. Groups of two to eleven C57BL/6J or CCR2 KO mice were infected ID with the indicated target doses of LVS ID; actual doses were confirmed by retrospective plate count and were within 15% of the stated target. Survival was monitored for at least 30 days, but no further deaths occurred after day 10. Data are pooled from eight individual experiments using different doses and numbers of mice per group. Overlapping lines are offset for clarity.
